# Serum IgE levels are a risk factor with prognosis of pediatric minimal change disease

**DOI:** 10.3389/fped.2023.1234655

**Published:** 2023-07-25

**Authors:** Tingting Han, Mei Xue, Yafei Guan, Tao Ju, Kaili Shi, Mengzhen Fu, Lili Jia, Chunlin Gao, Zhengkun Xia

**Affiliations:** ^1^ Department of Pediatrics, Jinling Hospital, Nanjing Medical University, Nanjing, China; ^2^ Department of Pediatrics, The First Affiliated Hospital of Nanjing Medical University, Nanjing, China; ^3^ Department of Pediatrics, Taizhou People's Hospital Affiliated to Nanjing Medical University, Taizhou, China; ^4^ Department of Pediatrics, Jinling Hospital, Nanjing, China

**Keywords:** minimal change disease, serum IgE, children, remission, relapse

## Abstract

**Background:**

Minimal change disease (MCD) is one of the most common primary glomerular disorders with high serum IgE levels. This study was aimed to investigate the clinical features of different serum IgE levels in pediatric MCD and evaluate the prognostic significance of serum IgE levels with regard to remission and relapse in pediatric cohort.

**Methods:**

This study enrolled 142 new-onset children diagnosed with biopsy-proven MCD from January 2010 to December 2021 at the Jinling Hospital in Nanjing, China. These cases were divided into three groups according to serum IgE levels. MCD patients’ demographics, clinical parameters, and follow-up data were collected and analyzed. The primary and secondary outcomes were defined as the time to the first complete remission (CR) and the first relapse.

**Results:**

The results manifested that 85.2% (121/142) of MCD children had high serum IgE levels (IgE > 90.0 IU/ml). A total of 142 patients were divided into the normal-, low-, and high-IgE groups based on the normal reference value level (90.0 IU/ml) and median serum IgE level (597.5 IU/ml). The high-IgE group had a significantly lower cumulative rate of the first CR (log-rank, *P *= 0.032) and a higher rate of the first relapse (log-rank, *P *= 0.033) than the normal-IgE and low-IgE groups. Multivariate Cox analysis showed that IgE ≥597.5 IU/ml was independently associated with the delayed first CR [hazard ratio (HR) = 0.566, 95% confidence interval (CI) = 0.330–0.972, *P *= 0.039] and the early first relapse (HR = 2.767, 95% CI = 1.150–6.660, *P *= 0.023).

**Conclusions:**

Serum IgE levels were an independent correlation factor for pediatric MCD-delayed remissions and early relapses.

## Introduction

Minimal change disease (MCD), which approximately accounts for 70%–90% in children >1 year of age and 10%–15% adults, respectively, in idiopathic nephrotic syndrome (INS), is one of the most common primary glomerular disorders. The morphologic feature of MCD is foot process effacement (1). Patients with MCD have a remarkably good response to corticosteroid therapy but are prone to relapse. The 40%–50% MCD in children, especially in children <5 years of age, have a frequently relapsing or steroid-dependent course, particularly during steroid tapering or soon after withdrawal, demanding second immunosuppressive agent therapy (2).

Numerous studies have reported a strong connection between INS and atopic disorders. Immunoglobulin E (IgE) is correlated with allergic reactions and elevated serum IgE levels are detected in patients with INS (3). Atopic disorders in MCD patients have varied widely, and increased IgE levels also are found in the absence of other clinical findings of atopy in MCD patients (4). High serum IgE levels may be correlated with poor responses to steroid therapy in INS children, poor prognosis, and frequent relapses. Serum IgE levels may not only reflect disease activity but may also implicate disease outcome in childhood of INS (5, 6). A recent study reported that MCD patients with high-IgE levels were observed to prolong first reach disease remission and prone to early relapses (7). Fewer relevant studies have been performed in pediatric and biopsy-proven MCD patients about correlation between serum IgE levels and prognosis of MCD. This study aims to explore the clinical features of patients with MCD according to serum IgE levels and evaluate prognostic significance of serum IgE levels with regard to remission and relapse in pediatric cohort.

## Methods

### Patients

Children (≤18 years) with MCD diagnosed by kidney biopsy were recruited from January 2010 to December 2021 in this retrospective study implemented by the Jinling Hospital. The indications for kidney biopsy in this cohort were as follows: (1) children first diagnosed with nephrotic syndrome (NS) at the age of ≥12 years; (2) children with steroid-resistant NS (SRNS); (3) children with atypical characteristics, involving macroscopic hematuria, not related to hypovolemia, hypertension, acute kidney injury (AKI), and rash that suggests glomerulonephritis; (4) steroid-sensitive NS (SSNS) patients evolving into secondary SRNS, steroid-dependent NS (SDNS), or frequent relapsing NS (FRNS) in the course of follow-up time. The study enrolled cases who met the following criteria: (1) 24-h urine protein to creatinine ratio (UP/CR) ≥2.0 g/g or ≥ 3+ on urine dipstick; (2) MCD patients with biopsy proven at any age between 1 and 18 years; (3) new-onset of disease or discontinuation of immunosuppressive therapy for more than 1 year. Individuals were excluded if they had any of the following conditions: (1) infectious diseases (e.g., hepatitis B or C, tuberculosis, AIDS); (2) individuals of connective tissue diseases, diabetes mellitus, malignant tumors; (3) drug-induced NS; (4) missing data on serum IgE levels at onset.

### Clinic data collect

All clinical data were obtained retrospectively from medical records. The baseline investigations were taken during the beginning of diagnosis. Follow-up data, including treatment, steroid response, time to the first complete remission (CR), and relapse, were collected and analyzed at 1, 2, 3, 6, and 12 months and longer follow-up. The last follow-up time was estimated as the last encounter in our follow-up system. The diagnosis of hypertension was based on the 2017 AAP Blood Pressure Clinical Practice Guidelines (8). eGFR was calculated using the Schwartz formula for children aged 16 years or less (9), and the CKD-EPI formula for children aged 17 years or older (10). The normal value of serum total IgE was ≤90 IU/ml and upper limit of detection was 1,000 IU/ml.

### Evaluation of treatment response

(1) SSNS was defined as CR within the 4 weeks standard dose of prednisone or prednisolone (PDN) (60 mg/m^2^/day or 2 mg/kg/day). (2) Steroid-resistant NS was defined as a lack of CR for at least 4 weeks of standard dose of steroids. (3) SDNS was defined as follows: (a) two consecutive relapses during corticosteroid therapy or within 15 days of discontinuing corticosteroid therapy; (b) infectious factors should be excluded at the same time; (c) when relapse occurs, patients would reach CR again once the dose of corticosteroid is increased (either at full dose or beyond the relapse dose). (4) Partial remission (PR) was defined as a decrease in 24-h UP/Cr to <2 but >0.2 g/g (or >20 and <200 mg/mmol) with a 50% reduction from its peak value, or the test result of the dipstick decreases by at least 1+, but it did not reach negative. (5) CR was defined as a decrease in 24-h UP/Cr ≤0.2 g/g (or 20 mg/mmol or negative or trace dipstick) on three or more consecutive occasions. (6) Total remission (TR) was the achievement of PR or CR. (7) Relapse was defined as an increase in UP/Cr to ≥2.0 g/g in cases with a 50% increase from its valley value or in urine dipstick to ≥3+ after the remission. (8) Frequent relapse was defined as ≥2 relapses per 6 months or ≥4 relapses per 12 months.

The primary and secondary endpoints of this cohort study were the first CR and the first relapse. When patients achieved the first CR after onset, we considered it as the first CR, and the time interval from treatment to the first day of CR would be recorded. Time to the first relapse was defined as the time interval from the TR to the occurrence of the first relapse.

### Statistical analysis

SPSS (version 26.0) and Graph Pad Prism 8.0 were used for statistical analysis in this cohort study. The Shapiro–Wilk test was performed whether the continuous variables conform to the normal distribution. Continuous variables were represented as mean ± SD deviation or median (interquartile range, Q1–Q3). Categorical data were presented as frequency (percentage) and analyzed using the *χ*^2^ test or Fischer's exact test. Survival rates were calculated using the Kaplan–Meier method. Parameters with a *P*-value <0.05 in the Cox univariate analysis were considered as confounding factors and were included in the multivariate Cox proportional hazard model. Results were presented as hazard ratios (HRs) and 95% confidence intervals (95% CIs). All data were tested two-sided, with *P *< 0.05 regarded as statistically significant.

## Results

### Baseline demographic and clinical features

**Figure 1:**
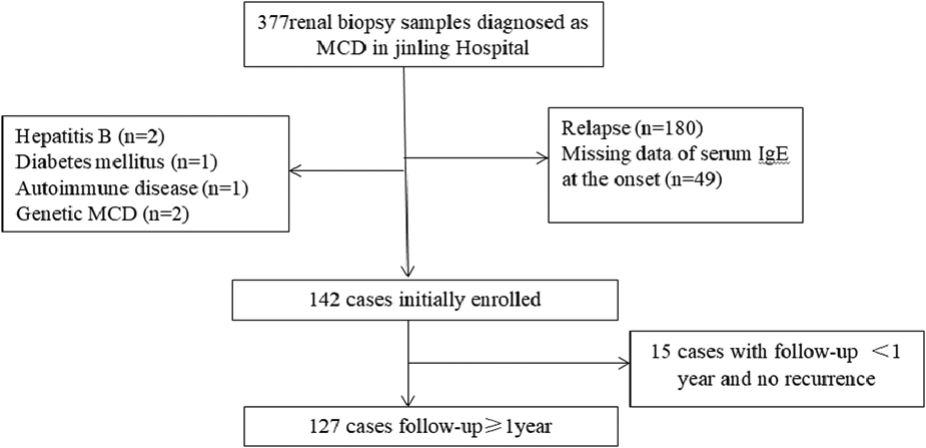
The flowchart shows 142 enrolled patients, 127 of whom had a follow-up more than 1 year.

As shown in Figure 1, from 2010 to 2021, 142 children were diagnosed with MCD based on renal biopsy in our center. Baseline demographic characteristics and clinical features of these patients at the beginning of diagnosis are listed in Table 1. Renal pathological biopsy was completed in 94.4% (135/142) of patients at onset, compared with 4.9% (7/142) in the course of follow-up treatment when patients underwent SRNS, SDNS, and FRNS. The median onset age was 15.0 (12.0–17.0) years, and the median age at biopsy was 15.0 (12.0–17.0) years. The median follow-up period of the patients was 40.2 (17.95–62.69) months. Upper respiratory infection (URI) was 30.8% (44/142) before occurrence of disease, acute gastroenteritis 12.6% (18/142), food allergy 0.7% (1/142), mosquito bite 0.7% (1/142), fatigue 3.5% (5/142), exposed to chemicals 1.4% (2/142), vaccination 1.4% (1/142), and gastroenteritis complicated with pneumonia 2.1% (3/142), while 46.9% (67/142) had no obvious predisposing cause. A total of 26 patients accounting for 18.3% of the total population had allergic history, including 2 cases of bronchial asthma, 5 cases of allergic rhinitis, 9 cases of atopic dermatitis, and 10 cases of other allergic condition such as food allergy and drug allergy. Only one patient presented eosinophilia at onset and the eosinophils returned to normal level with following treatment.

**Table 1:** Demographic characteristics and clinical feature.

Items	Overall *n* = 142	Normal-IgE group *n* = 21	Low-IgE group *n* = 50	High-IgE group *n* = 71	*P*
IgE level, IU/ml	597.5 (217.2 ≥ 1,000)	43.3 (20.0–62.9)	293.0 (204.0–393.0)	>1,000.0 (972.0 ≥ 1,000.0)	
Male, *n* (%)	110 (77.5%)	15 (13.6%)	38 (34.5%)	57 (51.8%)	0.663
Age of onset, years	15.0 (12.0–17.0)	15.0 (11.5–16.5)	15.0 (9.75–16.0)	15 (13.0–17.0)	0.341
Age at biopsy, years	15.0 (12.0–17.0)	15.0 (11.5–16.5)	15.0 (10.0–16.0)	15 (13.0–17.0)	0.108
BMI, kg/m^2^	20.8 (19–23.2)	20.3 (18.9–21.6)	20.5 (18.8–23.0)	21.5 (19.6–23.4)	0.341
Hypertension, *n* (%)	33 (23.2%)	6 (18.2%)	7 (21.2%)	20 (60.6%)	0.158
Allergy history	26 (18.3%)	5 (23.5%)	9 (6.3%)	12 (8.4%)	0.770
Bronchial asthma	2 (7.7%)	0	1 (50%)	1 (50%)	
Allergic rhinitis	9 (34.6%)	2 (22.2%)	4 (44.4%)	3 (33.3%)	
Atopic dermatitis	5 (19.2%)	1 (20.0%)	0	4 (80.0%)	
Other allergies	10 (38.5%)	2 (20.0%)	4 (40.0%)	4 (40.0%)	
eGFR, ml/min/1.73 m^2^	114.6 (97.6–138.6)	125.2 (97.9–147.8)	116.06 (94.5–145.6)	110.99 (97.5–133.8)	0.573
AKI, *n* (%)	10 (7%)	1 (10%)	0	9 (90%)*	**0**.**021**
Hematuria, *n* (%)	45 (31.6%)	6 (13.3%)	12 (26.7%)	27 (60%)	0.249
Urinary protein, g/day	5.9 (2.9–9.4)	5.0 (2.4–7.6)	5.4 (2.8–10.0)	6.3 (3.2–10.7)	0.64
Scr, μmol/L	60.1 (42.8–72.0)	58.30 (43.1–68.1)	57.2 (36.6–72.0)	64.8 (49.3–76.9)	0.091
Serum albumin, g/L	19.7 (16.7–22.5)	21.0 (17.3–22.7)	19.45 (16.9–22.5)	19.5 (16.2–22.6)	0.588
UA, μmol/L	379.0 (309.0–445.0)	362.0 (308.0–403.0)	376.0 (299.0–434.0)	381.5 (316.5–465.8)	0.223
TG, mmol/L	2.2 (1.4–3.4)	2.2 (1.3–4.5)	1.90 (1.4–3.4)	2.3 (1.4–3.2)	0.916
TC, mmol/L	10.5 (8.8–12.3)	10.1 (8.8–12.8)	10.7 (8.7–12.6)	11.0 (8.9–13.1)	0.923

BMI, body mass index; eGFR, estimated glomerular filtration rate; AKI, acute kidney injury; Scr, serum creatinine; UA, uric acid; TG, triglyceride; TC, total cholesterol.

* *P* < 0.05 vs. low-IgE group.

Bold value is statistically significant.

The median serum IgE level of MCD patients was 597.5 (217.25, >1,000) IU/ml and 85.2% (121/142) patients had high serum IgE levels (IgE > 90.0 IU/ml) at onset. Based on normal value and median of serum total IgE, we divided patients into three groups including 35.2% (50/142) in the low-IgE group (90–597.5 IU/ml) and 50% (71/142) in the high-IgE group (≥597.5–1,000 IU/ml), compared to 14.7% (21/142) in the normal-IgE group (≤90 IU/ml). There were no statistically significant differences in onset age, gender, body mass index (BMI), hypertension, allergic history, proteinuria levels, and hematuria cases (all *P *> 0.05). In the high-IgE group, the proportion of AKI patients was significantly higher than in low-IgE group (90% vs. 0%; *P* < 0.01).

### Blood routine test and immune indexes

The blood routine test and immune-related indexes of the patients during the initial treatment stage were collected and listed in Table 2. The high-IgE group had a higher level of serum IgA than in the normal-IgE and low-IgE groups [median, interquartile range (IQR): 2.04 (1.58–2.38) g/L vs. 1.71 (1.17–1.87) g/L and 1.75 (1.16–2.22) g/L; *P* = 0.04]. CD20% in the high-IgE group was higher than the normal-IgE and low-IgE groups [median (IQR): 16.7% (12.8%–23.6%) vs. 10.3% (6.4%–19.47%) and 11.0% (9.6%–18.9%), *P *= 0.038]. The high-IgE group had higher neutrophils (NE) [median (IQR): 4.29 (3.31–6.65) g/L vs. 3.45 (2.42–5.50) g/L; *P *= 0.035] and NE/Lymphocyte (LY) (neutrophil lymphocyte ratio, NLR) [median (IQR): 4.29 (3.31–6.65) g/L vs. 3.45 (2.42–5.50); *P *< 0.01) than low-IgE group that were shown in Supplementary Table S1.

**Table 2:** Laboratory date of immune and blood routine examination.

Items	Overall *n* = 142	Normal-IgE group	Low-IgE group	High-IgE group	*P*
Serum IgA, g/L	1.8 (1.3–2.3)	1.7 (1.2–1.9)	1.8 (1.2–2.2)	2.0 (1.6–2.4)	0.04
Serum IgM, g/L	1.5 (1.1–2.0)	1.3 (1.0–1.6)	1.6 (1.2–2.1)	1.5 (1.1–2.1)	0.124
Serum C3, g/L	1.1 (1.0–1.3)	1.1 (0.9–1.48)	1.2 (1.1–1.4)	1.1 (0.9–1.3)	0.428
Serum C4, g/L	0.2 (0.2–3.0)	0.3 (0.2–0.3)	0.3 (0.2–0.3)	0.2 (0.2–0.3)	0.231
CD20, %	14.8 (10.5–20.4)	10.3 (6.4–19.5)	11.0 (9.6–18.9)	16.7 (12.8–23.6)	**0**.**038**
CD3, %	70.1 (60.3–76.5)	77.9 (70.4–82.0)	73.9 (56.8–77.4)	68.4 (63.6–74.1)	0.175
CD4, %	39.1 (32.2–43.9)	41.5 (38.3–44.0)	38.5 (31.2–47.7)	38.8 (31.7–43.3)	0.595
CD8, %	27.8 (22.8–31.8)	31.0 (24.6–37.9)	25.6 (22.3–35.7)	27.1 (22.1–31.0)	0.432
CD4/CD8	1.5 (1.1–1.8)	1.5 (1.1–1.9)	1.2 (0.9–1.9)	1.5 (1.2–1.7)	0.836
NE, g/L	3.9 (2.8–5.9)	3.6 (2.5–5.9)	3.5 (2.4–5.5)	4.3 (3.3–6.7)*	**0**.**031**
LY, g/L	2.3 (1.7–3.2)	2.2 (1.60–3.60)	2.5 (2.1–3.5)	2.1 (1.5–3.2)	0.064
Eosinophilia, g/L	0.087 (0.006–0.181)	0.099 (0.005–0.173)	0.096 (0.013–0.185)	0.08 (0.000–0.180)	0.787
NE/LY(NLR)	1.6 (1.0–3.0)	1.1 (0.9–3.0)	1.2 (0.8–2.0)	2.0 (1.4–3.7)*	<**0**.**01**
PLT/LY	118.4 (87.0–161.9)	109.0 (79.4–144.8)	113.9 (80.1–136.4)	123.7 (94.1–187.3)	0.063
MO/LY	0.2 (0.1–0.2)	0.1 (0.1–0.2)	0.1 (0.1–0.2)	0.2 (0.1–0.2)	0.148

NE, neutrophil; LY, lymphocyte; PLT, blood platelet; NLR, neutrophil to lymphocyte ratio.

* *P* < 0.05 vs. low-IgE group.

Bold values are statistically significant.

### Treatment and response

The median follow-up duration is 36.5 months in the normal-IgE group, 38.7 months in the low-IgE group, and 41.3 months in the high-IgE group. Steroid therapy was administered to all 142 patients. Immunosuppressive agents (IA) other than steroids, such as tacrolimus (TAC) and cyclosporin A (CsA), mycophenolate mofetil (MMF), leflunomide (LEF), tripterygium glycosides (TG), cyclophosphamide (CTX), mizoribine (MZR), and rituximab (RTX) were applied in SR or SD patients. About 61.2% (87/142) patients received IA other than steroid therapy, which included 48.3% (42/87) cases in the high-IgE group, 13.8% (12/87) in the normal-IgE group, and 37.9% (33/87) in the low-IgE group. There were no statistically significant differences in the data collected above, as shown in Table 3. Throughout the whole course of the disease, 38.7% (55/142) patients only received one kind of IA, 45.7% (65/142) received two types of IA, and 15.4% (22/142) received at least three kinds of IA. At the last follow-up, 91.5% (130/142) patients achieved remission, including 88.0% (125/142) patients achieving CR and 3.5% (5/142) achieving PR. One patient (0.7%) was dead for cardiogenic shock and pulmonary edema after a follow-up of 28.63 months.

**Table 3:** Treatment and outcomes of minimal change disease.

Items	Normal-IgE group	Low-IgE group	High-IgE group	*P*
Follow-up, months	36.6 (15.3–67.6)	38.8 (20.0–71.6)	41.3 (18.9–56.0)	0.945
ACEI/ARB, *n* (%)	7 (33.3)	13 (26)	22 (30.9)	0.722
Steroid, *n* (%)	21 (100.0)	50 (100.0)	71 (100.0)	—
IA, *n* (%)	12 (57.1)	33 (66.0)	42 (59.2)	0.685
TAC/CsA	6 (28.6)	19 (38.0)	23 (32.4)	0.7
MMF	1 (5.0)	0	0	0.142
LEF	7 (35.0)	14 (28.0)	24 (33.8)	0.757
TG	1 (4.8)	6 (12.0)	6 (8.5)	0.602
CTX	0	2 (4.0)	0	0.248
RTX	2 (9.5)	4 (8.0)	3 (4.2)	0.57
Steroid response, *n* (%)				
Steroid-sensitive	14 (66.7)	25 (50.0)	38 (53.5)	0.677
Steroid-dependent	5 (23.8)	19 (38.0)	22 (31.0)	
Steroid-resistant	2 (9.5)	6 (12.0)	11 (15.5)	
Remission at the end of follow-up, *n* (%)				
NR	1 (4.8)	7 (14.0)	4 (5.6)	0.133
CR	20 (95.2)	43 (86.0)	62 (84.5)	
PR	0	0	5 (7.0)	

ACEI, angiotensin converting enzyme inhibitor; ARB, angiotensin receptor antagonist; IA, immunosuppressive agents; TAC, tacrolimus; CsA, cyclosporin A; MMF, mycophenolate mofetil; LEF, leflunomide; TG, tripterygium glycosides; CTX, cyclophosphamide; MZR, mizoribine; RTX, rituximab; NR, no remission; CR, complete remission; PR, partial remission.

**Figure 2:**
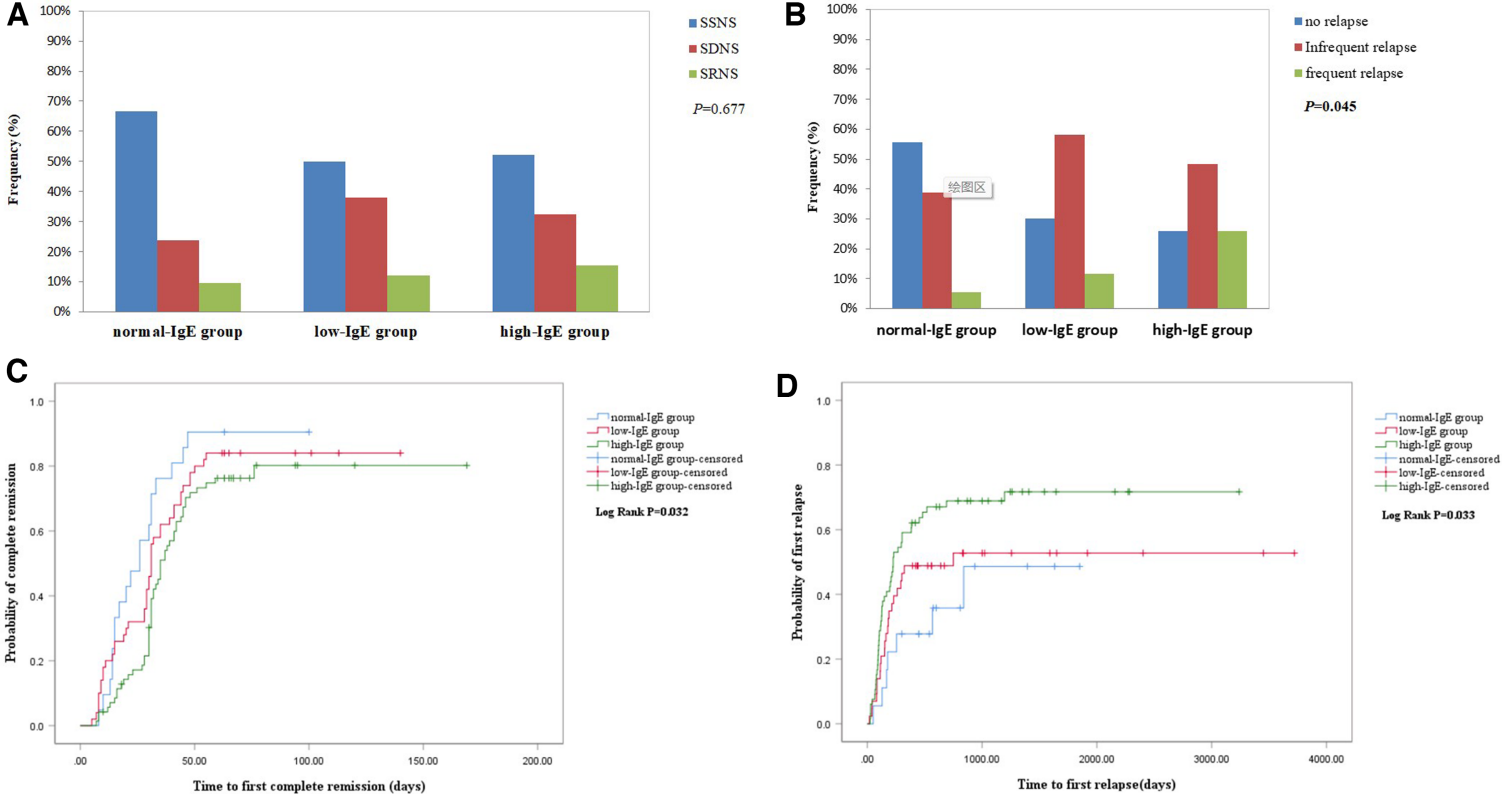
(**A**) Comparison of proportion of response to hormone therapy between the three groups. (**B**) Cumulative rate of the first complete remission. (**C**) Comparison of proportion of relapse between the three groups. (**D**) Cumulative rate of the first relapse.

In the normal-IgE group, proportions of SSNS, SDNS, and SRNS were 66.7% (14/21), 23.8% (5/21), and 9.5% (2/21), respectively, while proportions in the low-IgE group were 50% (25/50), 38% (19/50), and 12% (6/50) and in the high-IgE group were 53.5% (38/71), 31% (22/71), and 15.5% (11/71), as shown in Figure 2A. Unfortunately, there was no statistical difference between groups (*P *= 0.677). Median of serum IgE levels was 597 (208.5–1,000)  IU/ml in SSNS, 418.5 (206.25–1,000) IU/ml in SDNS, and 852 (329.0–1,000) IU/ml in SRNS, but there was no significantly difference between three groups. There was higher proportion of SSNS in the normal-IgE group than the low-IgE group and high-IgE group (66.7% vs. 50.0% and 53.5%), although a statistical difference was not reached. The IgE levels were recorded when 45cases of patients were in remission. IgE level in remission was lower than onset stage [595.0 (276–1,000) vs. 223 (112.3–669); *P* = 0.055].

### Predictors of the first CR

A total of 142 cases were followed up for more than 2 months and included to detect the predictors of the first CR.

As shown in Figure 2B, the Kaplan–Meier survival analysis showed that the high-IgE group had a lower cumulative rate of the first CR than the normal-IgE and low-IgE groups (log-rank, *P *= 0.032). Furthermore, we found a significant difference in the cumulative rate of the first CR between the high-IgE group and the normal-IgE group, which is presented in Supplementary Table S2. The average time to CR was 26.0 ± 21.34 days, 31.0 ± 28.32 days, and 35 ± 26.87 days in normal-IgE, low-IgE, and high-IgE groups, respectively. The 1-, 2-, 3-, and 6-month CR rates were 30.2% vs. 61.9% and 46.0%, 76.25% vs. 90.5% and 84.0%, 80.2% vs. 90.4% and 84.0%, 80.2% vs. 90.4% and 84.0% for the high-IgE group vs. normal-IgE group and low-IgE group, respectively. The results of the univariate Cox regression analysis showed that in the high-IgE group (HR = 0.516, CI = 0.4–1.187, *P *= 0.014), SD (Reference = SS; HR = 0.643, 95% CI = 0.431–0.959, *P *= 0.03), SR (Reference = SS; HR = 0.162, 95% CI = 0.077–0.342, *P < *0.01), AKI (HR = 0.399, 95% CI = 0.162–0.981; *P* = 0.045), and eosinophils count (HR = 4.58, 95% CI = 1.19–7.634, *P *= 0.027) were associated with the first CR. The result of the multivariate Cox regression analysis is listed in Table 4. IgE ≥ 597.5 (HR = 0.566, 95% CI = 0.33–0.972, *P *= 0.039) and SR (Reference = SS; HR = 0.158, 95% CI = 0.071–0.352, *P < *0.01) were independently associated with the first CR within 2 months.

**Table 4:** Risk factors for first CR analyzed with Cox univariate and multivariate.

Items.	Cox univariate analysis	*P*	Cox multivariate analysis	*P*
	HR (95% CI)		HR (95% CI)	
Age at onset, years	1.006 (0.969–1.045)	0.740	—	—
Sex, male	0.956 (0.609–1.500)	0.844	—	—
Hypertension	1.198 (0.777–1.847)	0.414	—	—
eGFR, ml/min/1.73 m^2^	0.999 (0.995–1.003)	0.650	—	—
AKI	0.399 (0.162–0.981)	**0**.**045**	0.702 (0.276–1.786)	0.458
Steroid response, *n* (%)				
SD (reference to SSNS)	0.643 (0.431–0.959)	**0**.**03**	0.710 (0.467–1.08)	0.11
SR (reference to SSNS)	0.162 (0.077–0.342)	**<0**.**01**	0.158 (0.071–0.352)	<**0.01**
Proteinuria, g/day	0.979 (0.940–1.019)	0.303	—	—
Serum albumin, g/L	1.007 (0.974–1.041)	0.684	—	—
eosinophilia	4.580 (1.190–7.634)	**0**.**027**	3.279 (0.433–7.641)	0.108
IgE levels				
low-IgE group (reference to normal)	0.689 (0.400–1.187)	0.18	0.753 (0.227–1.144)	0.316
High-IgE group (reference to normal)	0.516 (0.305–0.874)	**0**.**014**	0.566 (0.330–0.972)	**0.039**

eGFR, estimated glomerular filtration rate; AKI, acute kidney injury; SD, steroid-dependent; SR, steroid-resistant; SS, steroid-sensitive.

### Predictors of the relapse

A total of 127 patients with a follow-up of more than 1 year were included to detect the predictors of the first relapse.

In the normal-IgE group, proportions of no relapse (NR), infrequent relapse (IFR), and frequent relapse (FR) were 55.5% (10/18), 38.8% (7/18), and 5.5% (1/18), respectively, whereas that in the low-IgE group were 30.23% (13/43), 58.13% (25/43), and 11.6% (5/43) and in the high-IgE group were 25.7% (17/66), 48.4% (32/66), and 25.7% (17/66), respectively. As shown in Figure 2C, the high-IgE group had a significantly higher proportion of patients with FR (*P *= 0.045).

Figure 2D showed that the high-IgE group had a significantly higher cumulative rate of the first relapse (log-rank, *P *= 0.033). The mean time to first relapse in normal-, low-, and high-IgE groups were 18.45 ± 17.2 months, 13.13 ± 28.17 months, and 7.50 ± 22.23 months, respectively. The 3-month, 6-month, 1-year, and 2-year relapse rates were 18.18% vs. 5.5% and 13.95%, 40.90% vs. 22.2% and 30.23%, 59.09% vs. 27.77% and 48.83%, and 68.89% vs. 35.80% and 52.77% for the high-IgE group vs. normal-IgE group and low-IgE group, respectively. The univariate Cox regression analysis showed that high-IgE group (Reference = normal-IgE group; HR = 2.38, 95% CI = 1.074–5.277, *P *= 0.033) and serum albumin (HR = 0.929, 95% CI = 0.882–0.979, *P *< 0.01) were associated with the first relapse within 1 year. Other significant parameters in the univariate analysis included age at onset, SD, SR, and triglyceride (TG). As shown in Table 5, the Cox multivariate regression model revealed that in the high-IgE group (Reference = normal-IgE group; HR = 2.767, 95% CI = 1.15–6.66, *P *= 0.023), serum albumin (HR = 0.945, 95% CI = 0.896–0.997, *P *= 0.037) and TG (HR = 1.098, 95% CI = 1.014–1.190, *P *= 0.021) were independent risk factors with the first relapse within 1 year.

**Table 5:** Risk factors for first relapse analyzed with Cox univariate and multivariate.

Items	Cox univariate analysis	*P*	Cox multivariate analysis	*P*
	HR (95% CI)		HR (95% CI)	
Age at onset, years	0.944 (0.901–0.989)	**0**.**016**	1.017 (0.960–1.078)	0.561
Sex, male	1.067 (0.628–1.814)	0.81	—	—
Hypertension	0.341 (0.084–1.390)	0.133	—	—
eGFR, ml/min/1.73 m^2^	1.001 (0.995–1.008)	0.663	—	—
AKI	2.385 (0.957–5.947)	0.062	—	—
Steroid response, *n* (%)				
SD (reference to SSNS)	2.852 (1.711–4.755)	**<0**.**01**	2.865 (1.592–5.157)	**<0**.**01**
SR (reference to SSNS)	4.346 ( 2.095– 9.014)	**<0**.**01**	4.337 (1.858–10.120)	**<0**.**01**
Proteinuria, g/day	1.001 ( 0.951–1.054)	0.968	—	—
Serum albumin, g/L	0.929 (0.882–0.979)	**<0**.**01**	0.945 ( 0.896–0.997)	**0**.**037**
TG	1.114 (1.047–1.185)	**<0**.**01**	1.098 (1.014–1.190)	**0**.**021**
IgE levels				
Low-IgE group (reference to normal)	1.524 ( 0.651–3.568)	0.332	1.713 (0.654–4.488)	0.273
High-IgE group (reference to normal)	2.380 (1.074–5.277)	**0**.**033**	2.767 (1.150–6.660)	**0**.**023**

eGFR, estimated glomerular filtration rate; AKI, acute kidney injury; SD, steroid-dependent; SR, steroid-resistant; SS, steroid-sensitive; TG, triglyceride.

## Discussion

This retrospective study assessed the serum IgE levels and correlated risk indicators in 142 Chinese children with MCD. So far, this study is the large-scale one in children with MCD and found that serum high levels of IgE were an independent risk factor delayed remission and an early relapse.

Researchers had noted that patients with INS had a higher incidence of allergic diseases. Several patients had relapses of INS during asthma attacks or had undergone spontaneous remission of respiratory symptoms with hyposensitization therapy (3). A high proportion of patients with INS had higher allergen-specific IgE levels. More than 50% of adult patients with MCD had serum IgE higher than 2 SD above normal level (11). Schulte-Wissermann et al. showed that the IgE levels of children with MCD were significantly higher and the high levels IgE in the relapse phase decreased only slightly during remission (12). In our study, 85.2% patients had high serum IgE levels at onset and only one patient induced INS on account of seafood. Elevated IgE levels indicated the greater prevalence of allergy history, although there were no significant differences in allergy history between groups in our study. Meanwhile, difference in eosinophil count was not observed between groups in our study and only one patient had obvious eosinophilia at onset but the eosinophils returned to normal level with following treatment. We assessed the serum IgE levels while children were in remission, which were lower than onset stage.

There was a significantly higher occurrence rate of AKI in the high-IgE group of MCD, which was similar to a previous study (13). Previous research studies showed that in patients with MCD, CD23+ cells, CD20+ CD23+ cells, and CD23/CD20 ratio were significantly increased and, furthermore, CD23/CD20 ratio in MCD was significantly correlated with the serum IgE level (14). Our study indicated that CD20 percentage was remarkably higher in the high-IgE group. Elevated IgE levels in MCD may reflect the activation of B cells and T cells. NEs as the most frequent type of inflammatory cells are responsive to infectious agents. It was reported that NE and TH2 could generate an interaction effect and reduce T-cell function (15). NLR can be used to evaluate the systemic inflammation as it could be calculated easily. NLR has a prognostic value in various cancers, cardiovascular diseases, respiratory diseases, severe infection, and so on (16). Recent studies indicated that NLR was significantly higher in children with allergic rhinitis and recurrent wheezing (17, 18). In our research, we found that NE and NLR were significantly increasing in the high-IgE group than other groups. With regard to immune cell trafficking, there is a probable explanation for increased NLR in peripheral blood here. During infection or exacerbation of inflammation, tissue NE was found to be increased and released from these compartments into the blood stream. Meanwhile, lymphocytes also conveyed to compartments such as the spleen and lymph nodes, where they can better respond to the immune system (19). In our study, NLR as an integrated reflection of two distinctive yet complementary immune pathways partly demonstrated immune dysregulation in MCD (20).

Previous studies indicated that serum IgG level combined with serum IgE level could be served as prognostic clinical indicators of SSNS (21). It had been shown that the serum IgE levels were higher in SSNS and FRNS (22). Youn et al. incorporated 32 SSNS into their study and reported that the median time to the initial remission was 8.5 days in the normal-IgE group and 12.5 days in the high-IgE group, and the median duration of total steroid therapy was longer in the high-IgE group than the normal-IgE group (5). We observed a delayed remission for MCD children in the high-IgE group, which was in accordance with previous studies. However, there was no significant difference of SSNS proportion between in each group. Patients in the high-IgE group had a higher recurrence rate and FR rate than those in the normal-IgE group. Research had shown that 70.0% (14/20) of cases with high IgE and 16.7% (2/12) cases with normal IgE in SSNS were observed to have recurrence (5). Serum IgE was persistently and significantly higher in FRNS than IFRNS (23). In our study, it was found that the time to first relapse of MCD children in the high-IgE group was significantly shorter, which was consistent with other studies’ results (7, 24). In addition, a higher percentage of MCD patients experienced FR in the high-IgE group. Our multivariate Cox analysis showed that IgE ≥597.5 IU/ml was independently associated with the delayed first CR and the early first relapse. In order to conveniently implement in clinical, we advised IgE ≥600 IU/ml as risk factor with prognosis of MCD.

The immune disordered Th cells in MCD could be subdivided into Th0, Th1, Th2, and other different cell subsets according to the different cytokines which they secrete. It was observed that the imbalance of Th1/Th2 cells was common in INS and pathogenesis of INS was closely related to the activation of Th2 (25). Activation of Th2 cells resulted in the releasing of IL-13 and IL-4 cytokines and then stimulating B cells to produce serum IgE (26). Another way of generating IgE was surface antigen CD40 and T-cell surface CD40 ligand binding (27). IgE could stimulate mast cells to degranulate and release vasoactive substances that can act on the glomerular capillaries that could produce a large amount of proteinuria and lead to the onset of INS (26). B cells were the main cells that produce immunoglobulins and serum IgE levels were increased in cases of children with FRNS. That accounted for partly that B cells played a role in the pathogenesis of INS (28).

## Conclusion

In summary, this study found that serum IgE levels are an independent risk factor for delayed remission and relapse in pediatric MCD patients. These findings agree with the potential theory that the immune system is involved in the progression of MCD. Nonetheless, a frontier research study is needed to verify the function of IgE on MCD pathogenesis and theranostics.

## Data Availability

The original contributions presented in the study are included in the article/**Supplementary Material**, further inquiries can be directed to the corresponding authors.
