# Supplementary material for: Serum IgE levels are a risk factor with prognosis of pediatric minimal change disease
Source: Front Pediatr. 2023 Jul 25;11:1234655. doi: 10.3389/fped.2023.1234655 (PMC10407802; doi:10.3389/fped.2023.1234655)
Supplement: Supplementary file 1 [file Table1.docx]

| Items/P | normal–IgE group | low–IgE group | High-IgE group |
| --- | --- | --- | --- |
| **AKI,%** | 1（10%） | 0 | 9（90%） |
| normal–IgE group | - | 0.296 | 0.447 |
| low–IgE group | 0.296 | - | **0.01** |
| High-IgE group | 0.447 | **0.01** | - |
| **IgA,g/L** | 1.7（1.2-1.9） | 1.8（1.2-2.2） | 2.0（1.6-2.4） |
| normal–IgE group | - | 1.0 | 0.076 |
| low–IgE group | 1.0 | - | 0.203 |
| High-IgE group | 0.076 | 0.203 | - |
| **CD20,%** | 10.3% (6.4-19.5) | 11.0 (9.6-18.9) | 16.7（12.8-23.6） |
| normal–IgE group | - | 1.0 | 0.131 |
| low–IgE group | 1.0 | - | 0.141 |
| High-IgE group | 0.131 | 0.141 | - |
| **NE,g/l** | 3.6（2.5-5.9） | 3.5（2.4-5.5） | 4.3（3.3-6.7） |
| normal–IgE group | - | 1.0 | 0.409 |
| low–IgE group | 1.0 | - | **0.035** |
| High-IgE group | 0.409 | **0.035** | - |
| **NLR** | 1.1 (0.9-3.0) | 1.2 (0.8-2.0) | 2.0 (1.4-3.7) |
| normal–IgE group | - | 1.0 | 0.14 |
| low–IgE group | 1.0 | - | **＜0.01** |
| High-IgE group | 0.14 | **＜0.01** | - |

**Supplemental table1 Comparison of laboratory results between two groups**

**Supplemental table 2 Comparison of survival analysis between two groups**

| Items/P | normal–IgE group | low–IgE group | High-IgE group |
| --- | --- | --- | --- |
| **first CR** |  |  |  |
| normal–IgE group | - | 0.218 | **＜0.01** |
| low–IgE group | 0.218 | - | 0.151 |
| High-IgE group | **＜0.01** | 0.151 | - |
| **first relapse** |  |  |  |
| normal–IgE group | - | 0.33 | **0.027** |
| low–IgE group | 0.33 | - | 0.081 |
| High-IgE group | **0.027** | 0.081 | - |
